# 2-Methoxyestradiol and Its Combination with a Natural Compound, Ferulic Acid, Induces Melanoma Cell Death via Downregulation of Hsp60 and Hsp90

**DOI:** 10.1155/2019/9293416

**Published:** 2019-10-02

**Authors:** Anna Kamm, Paulina Przychodzeń, Alicja Kuban–Jankowska, Antonella Marino Gammazza, Francesco Cappello, Agnieszka Daca, Michał A. Żmijewski, Michał Woźniak, Magdalena Górska–Ponikowska

**Affiliations:** ^1^Department of Medical Chemistry, Medical University of Gdansk, Gdansk 80-211, Poland; ^2^Euro-Mediterranean Institute of Science and Technology, Palermo, Italy; ^3^Department of Biomedicine, Neurosciences and Advanced Diagnostics (BiND), University of Palermo, 90127 Palermo, Italy; ^4^Department of Pathology and Rheumatology, Medical University of Gdansk, Gdansk 80-211, Poland; ^5^Department of Histology, Medical University of Gdansk, Gdansk 80-211, Poland; ^6^Department of Biophysics, Institute of Biomaterials and Biomolecular Systems, University of Stuttgart, Stuttgart, Germany

## Abstract

Melanoma is an aggressive type of skin cancer with one of the highest mortality rates. Notably, its incidence in the last few decades has increased faster than any other cancer. Therefore, searching for novel anticancer therapies is of great clinical importance. In the present study, we investigated the anticancer potential of 2-methoxyestradiol, potent chemotherapeutic, in the A375 melanoma cellular model. In order to furthermore evaluate the anticancer efficacy of 2-methoxyestradiol, we have additionally combined the treatment with a naturally occurring polyphenol, ferulic acid. The results were obtained using the melanoma A375 cellular model. In the study, we used MTT assay, flow cytometry, and western blot techniques. Herein, we have evidenced that the molecular mechanism of action of 2-methoxyestradiol and ferulic acid is partly related to the reduction of Hsp60 and Hsp90 levels and the induction of nitric oxide in the A375 melanoma cell model, while no changes were observed in Hsp70 expression after 2-methoxyestradiol and ferulic acid treatment separately or in combination. This is especially important in case of chemoresistance mechanisms because the accumulation of Hsp70 reduces induction of cancer cell death, thus decreasing antitumour efficacy.

## 1. Introduction

Melanoma is an agressive type of skin cancer with one of the highest mortality rates, while its incidence in the last few decades has increased faster than any other cancer [1]. Although there has been tremendous progress in the treatment of melanoma patients in recent years, and in over the last 7 years the US Food and Drug Administration (FDA) has authorized many antimelanoma drugs, the ideal treatment is still not clearly defined and remains the subject of great debates [2].

While being an integral evaluation criterion of graduation of melanoma for many years, Clark's level is no longer recommended as it is not an independent prognostic factor. Histological features such as tumour thickness, but also rate of mitosis, are crucial for prognosis and determination of the stage of melanoma [3]. The evidence-based analysis that led to the development of recommendations for the assessment of melanoma progression was based on the updated database of the US Cancer Staging Manual (AJCC). The Melanoma Evaluation Committee recommended that the mitotic rate should be determined by the “hotspot” method and expressed as the number of mitoses per square millimeter of the primary tumour [4]. The Melanoma Evaluation Committee recommended that the mitotic rate should replace Clark's level as the main criterion for determining T1b melanoma [5]. Moreover, it is enormously vital to constantly strive to explore knowledge about substances that can increase the effectiveness of cancer therapies. A larger understanding of the molecular mechanisms of potential drugs can lead to creating new or developing existing therapies that take into account the individual physiological profile of the patient.

Anticancer agent that may be effective in treatment of melanoma is 2-methoxyestradiol (2-ME), which is a natural compound, a metabolite of 17*β*-estradiol, and a hormone of both women and men [6]. 2-ME is a monomethyl ether of 2-hydroxyestradiol formed in the reaction catalyzed by catechol-O-methyltransferase (COMT). Its physiological level in the blood serum ranges from 30 pM up to 30 nM during pregnancy [6], while pharmacological relevant concentrations involve micromolar concentrations [7]. Induction of nitro-oxidative stress is involved in antitumour activity of 2-ME against various cancer cellular models. In our previous studies, we evidenced that 2-ME, at both physiological and pharmacological relevant concentrations, increases the nuclear fraction of neuronal nitric oxide synthase (nNOS) in osteosarcoma 143B cells. Thus, we suggested nNOS as a molecular messenger of 2-ME. Induction of nNOS via 2-ME increased production of nitric oxide leading to DNA strand breaks and eventually cell death [8]. Another anticancer mechanism of 2-ME revealed by our team is regulation of mitochondrial biogenesis and inhibition of the activity of succinate dehydrogenase complex in osteosarcoma 143B cells [9].

The effectiveness of 2-ME has been demonstrated *in vitro* in many cancers, including lung cancer, breast cancer, colorectal cancer, and pancreatic cancer [10–16]. Currently, 2-ME trade name PANZEM is in the second phase of clinical trials in the treatment of kidney, prostate, ovarian, and carcinoid tumours with high metastatic potential [7, 17–26]. Notably, 2-ME seems to be cytotoxic towards melanoma cells in both *in vitro* and *in vivo* models [12, 27–29]. It is further hypothesized that 2-ME specifically kills cancer cells without affecting normal cells [30].

**Figure 1:**
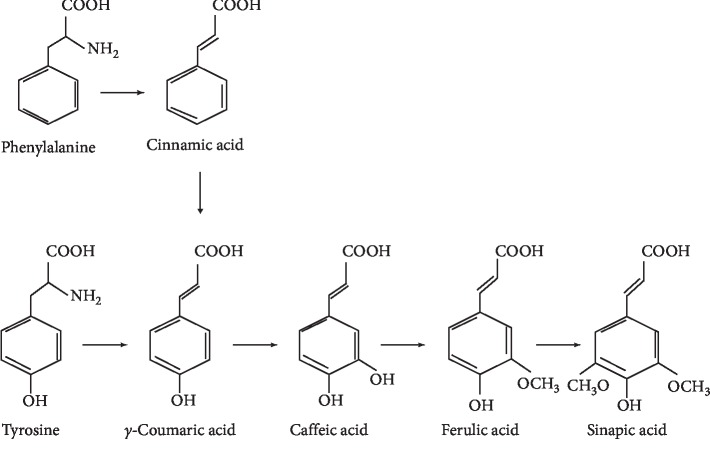
The synthesis pathway of hydroxycinnamic acids in plants (Castelluccio i wsp., 1995).

In the current study, we combined 2-ME with a natural compound, ferulic acid (FA). FA belongs to the group of hydroxycinnamic acids found in plant tissues [31] (Figure 1). FA is a phenolic compound that possesses three characteristic structural domains that may contribute to the ability to reduce free radicals [32]. The antioxidant properties of FA depend on its chemical structure [33]. FA, due to the phenolic structure and the unsaturated side chain, may easily form a resonant-stabilized phenoxyl radical, which is responsible for its strong antioxidant activity [34]. The health benefits of using phenolic compounds, such as FA, attract the attention of many researchers due to their antioxidant potential. Antitumor activity of polyphenols includes antiproliferative and proapoptotic effects in tumour cells [35]. Phenolic acids of plant origin, like FA with strong antioxidant activity, have received special attention as potential tumour inhibitors [36].

Notably, major heat shock proteins, such as Hsp90, Hsp70, and Hsp60, may be considered as biomarkers for cancer diagnosis and prognosis, as well as efficacy of anticancer therapies [37–42]. These Hsps are also implicated in cancerogenesis and further progression of melanoma [43–45]. Therefore, herein we address the question about the role of major Hsps in efficacy of supportive anticancer treatment of FA separately and in combination with a potent, anticancer agent, 2-ME, in the A375 melanoma cellular model.

## 2. Materials and Methods

### 2.1. Cell Culture

Human melanoma A375 cells (CRL-1619) were purchased from the American Type Culture Collection (Manassas, VA, USA). The cells were cultured in Dulbecco's modified Eagle's medium (DMEM) supplemented with 10% fetal bovine serum (FBS) (both Sigma-Aldrich; Merck KGaA) and 1% penicillin/streptomycin in an incubator with 5% CO_2_ at 37°C.

### 2.2. Experimental Design: Cell Treatment

In the study we used the A375 human melanoma cell model. First of all, the A375 cells were seeded in the standard medium at appropriate densities on the plates according to the specific experimental design 24 h before the treatment. The treatments were performed in DMEM containing 1% charcoal-stripped FBS and 1% antibiotic cocktail (Sigma-Aldrich, Poland). Charcoal-stripped FBS is used to elucidate the effects of hormones in various *in vitro* systems.

Subsequently, the A375 cells were treated with 2-ME separately or in combination with FA for 24 hours or 8 hours according to the experimental design. Based on previous research, 10 *μ*M 2-ME was used [8, 46]. While, based on MTT results, we chose 1 mM FA for further studies. Following the incubation, the cells underwent procedure according to the specific experimental design described below.

In order to avoid the impact of the solvents, for the further studies, control cells were treated with an equal volume of the solvent used to prepare 2-ME and FA solutions. The final concentration of solvents in the incubation medium was less than 0.1%.

### 2.3. Cell Viability/Cell Proliferation Assay (MTT Assay)

A375 melanoma cells were seeded into a 96-well plate at a density of 10,000 cells per well. After 24 hours, the cell culture medium was removed and the cells were treated with serial dilutions of FA within the concentration range between 1 mM and 31.25 *μ*M. Based on the results, for further studies 1 mM FA was chosen. Consequently, the cells were treated with 1 mM FA and 10 *μ*M 2-ME separately or in combination for 24 hours. Solvent-treated A375 melanoma cells were considered as the control (100% of cell viability).

After the appropriate incubation time, 0.5 mg/ml of 3-[4,5-dimethylthiazol-2-yl]-2,5-diphenyltetrazolium bromide (MTT) was added (Sigma-Aldrich, Poland). The plates were incubated at 37°C for 4 hours, and the supernatant was removed after centrifugation (700 ×*g* for 10 min). Finally, 100 *μ*l of DMSO (Sigma-Aldrich, Poland) was added to dissolve the formazan crystals. Absorbance at 570 nm was read using a microplate reader (BioTek Instruments, Inc., USA). The data are presented as a percent of control. Each experiment was carried out at least three times.

### 2.4. Determination of the Nitro-Oxidative Stress Pool by Flow Cytometry

A375 cells were seeded into 6-well plates at the density of 300,000 cells per well. Subsequently, A375 cells were treated with 1 mM FA and 10 *μ*M 2-ME separately or in combination for 8 hours. Eight hours incubation time for nitro-oxidative stress was based on our previous results [8, 46].

The level of oxidative stress was determined by the fluorescence intensity of 2,7-dichlorofluorescein diacetate (DCF-DA), and DCF was added at 10 *μ*M final concentration 30 minutes before the end of incubation time. DCF does not show fluorescent properties until the oxidation reaction is carried out in presence of free radicals.

The cells were detached from the plates with trypsin, collected, and centrifuged (1200*g* for 5 minutes). Washed twice with phosphate buffered saline (PBS; 137 mM NaCl, 2.7 mM KCl, and 4.3 mM Na2HPO4, pH 7.4), suspended in PBS, and then analyzed by flow cytometry. The nitric oxide level was determined using a 4,5-diaminofluorescein diacetate (DAF-DA) detector. A LSR II flow cytometer (Becton Dickinson, USA) equipped with FACSDiva software was used. The entire procedure was carried out on ice. Then 30,000 cells were counted and analyzed by flow cytometry (BD FACScan) with a dye spectrum filter (excitation *l* = 495 and *l* = 530). The results were analyzed using Cyflogic software, version 1.2.1. The procedure was repeated at least 3 times to ensure repeatability of results.

### 2.5. Analysis of Apoptosis and Necrosis by Flow Cytometry

Analysis of the level of apoptosis and necrosis was performed by means of flow cytometry. Briefly, A375 cells were seeded in 6-well plates at a density of 300,000 cells per well. After 24 hours, the cells were treated with 1 mM FA and 10 *μ*M 2-ME separately or in combination for 24 hours. The cells were then trypsinased and then harvested by centrifugation at 1200*g* for 7 minutes. The samples were washed 3 times with ice-cold PBS. The cells were then incubated with annexin V and PI for 15 minutes at room temperature. The whole procedure except incubation with annexin V and PI was carried out on ice. The cells were then counted at 30,000, and the fluorescence signals of annexin V and PI conjugate were detected in fluorescence intensity channels FL1 and FL3 (BD FACScan). The results were analyzed using Cyflogic software, version 1.2.1. The procedure was repeated at least 3 times to ensure repeatability of results.

### 2.6. Analysis of Hsp 70, 60, and 90 Protein Levels by Western Blot Technique

The level of Hsp 70, 60, and 90 proteins and *β*-actin were determined by western blot technique. After 24 hours, the cells were treated with 1 mM FA and 10 *μ*M 2-ME separately or in combination for 24 hours. Then, the cells were harvested and centrifuged. The pellets were washed 3 times with PBS (Sigma-Aldrich, Poland) and then suspended in RIPA buffer (Sigma-Aldrich, Poland) and a cocktail of protease inhibitors (Calbiochem, Germany). Protein concentration was determined using the Bradford reagent [Bradford, 1976]. Afterwards, samples containing 100 *μ*g of protein were mixed with Laemmli loading buffer (Sigma-Aldrich, Poland) and incubated at 95°C for 10 min. The proteins were separated on a 7–20% gradient of polyacrylamide gel (GE Healthcare, Poland) by electrophoresis. The separated proteins were transferred to a methanol-activated PVDF membrane in TBE buffer (90 mM Tris, 90 mM boric acid, and 1 mM EDTA, pH 8) using a semi-dry transfer device (250 mA, 63 V, and 45 minutes) (GE Healthcare, Poland). Then, after 1 hour of blocking in 5% nonfat milk in TBS-T (0.5% Tween20, 20 mM Tris-HCl, pH 7.4, and 0.5 M NaCl), the membranes were incubated with primary antibodies overnight at 4°C. The Hsp90 beta antibody (catalog number ab80159) and Hsp70 antibody [EP1007Y] purchased from Abcam (catalog number ab45133), and Hsp60 antibody (H-1) (catalog number sc-13115) and beta-actin (catalog number sc-47778) purchased from Santa Cruz Biotechnology were used in the study. After incubation time, the membranes were washed 3 times for 5 minutes in TBS-T and then incubated with horseradish peroxidase (HRP) conjugated secondary antibodies (1 : 50,000 dilution in TBS-T) for 1 hour at room temperature. Visualization was performed using chemiluminescence enhanced with a luminol reagent (chemiluminescence blotting, GE Healthcare, Poland) according to the manufacturer's protocol. The signal was read using ImageQuant LAS 500 (GE Healthcare, Poland). Protein levels were quantified using densitometry analysis by the Quantity One program. The results were normalized to β-actin. Each experiment was carried out at least three times.

### 2.7. Statistical Analysis of the Obtained Results

The results are represented by the mean ± SD of at least three independent experiments. Differences between control and treated samples were assessed by means of one-way analysis of variance (ANOVA) with a post hoc test using Tukey's multiple comparison test. A *p* value less than 0.01 was considered to be equivalent to statistical significance. Data were analyzed using GraphPad Prism (GraphPad Software, Inc., version 6, USA).

## 3. Results

### 3.1. Antiproliferative Effect of FA and 2-ME in the Melanoma A375 Cellular Model

**Figure 2:**
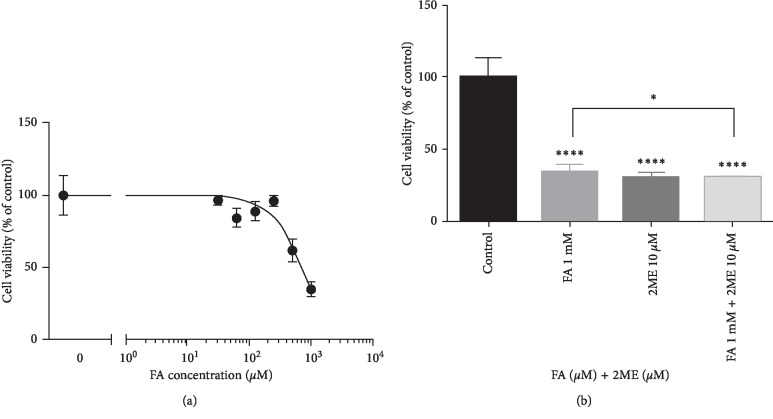
(a) The A375 cell viability graph after incubation with FA within the concentration range between 1 mM and 31.25 *μ*M for 24 hours. (b) The viability of melanoma A375 cells is inhibited after treatment with 10 *μ*M 2-ME, 1 mM FA, and combination of both for 24 hours. The cell viability was determined by MTT assay. Values are the mean ± SE of six independent experiments (*N* = 6 repeats). ^*∗*^*p* < 0.01 and ^*∗∗∗∗*^*p* < 0.00001 vs. control. Statistical significance was determined by a one-way ANOVA analyses followed by Tukey's multiple comparison test and unpaired *t* test.

First of all, we addressed the question about antiproliferative efficacy of FA in A375 cells by means of MTT assay. The antiproliferative potential of FA was evaluated by 24-hour treatment of A375 cells with serial dilutions of FA within the concentration range between 1 mM and 31.25 *μ*M (Figure 2(a)). The percentage of viable cells in samples was calculated in comparison to control A375 cells, which viability was assumed to be 100%. Based on the survival curves obtained by the GraphPad Prism Software, the calculated EC50 (50% decrease in the viability of the treated cells) concentration was equal to 701.9 *μ*M.

Therefore, for further studies, a representative concentration of 1 mM FA was chosen. The next goal of the study was to determine the efficacy of combined treatment of FA with a potent anticancer agent, 2-ME, in the melanoma A375 cellular model. The concentration of 10 *μ*M of 2-ME was chosen as representative, corresponding to the pharmacological concentration range, based on previous studies [8, 46]. As presented in Figure 2(b), we did observe statistical significant correlation between combined treatment of FA and 2-ME as compared to separate treatments.

### 3.2. Effect of Combined and Separate Treatment with FA and 2-ME on Induction of Melanoma A375 Cell Death

In order to further explore the anticancer efficacy and interaction between 2-ME and FA in A375 cells, we next determined the impact of the compounds on the induction of cell death.

**Figure 3:**
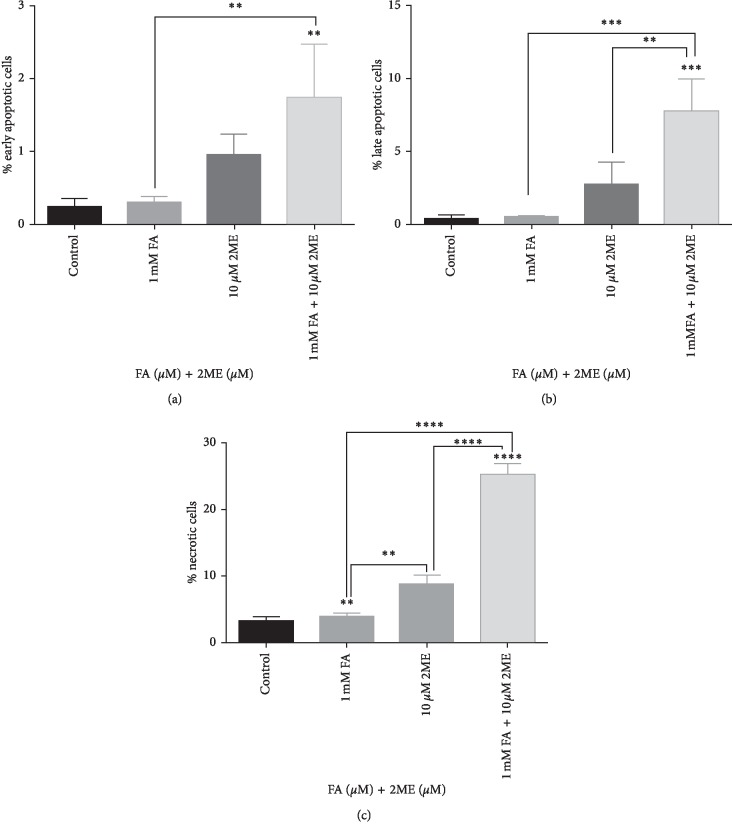
(a) Total cell levels in the early apoptosis phase after 24 hours incubation of A375 line cells with 10 *μ*M 2-ME and 1 mM FA separately or in combination. (b) Total cell level in the late phase of apoptosis after 24 hours incubation of A375 line cells with 10 *μ*M 2-ME and 1 mM FA separately or in combination. (c) Total cell level in the necrotic phase after 24 hours incubation of A375 with 10 *μ*M 2-ME and 1 mM FA separately or in combination. Values are the mean ± SE from three independent experiments. No error bar means the thickness of the line is greater than the error. ^*∗*^*p* < 0.01 compared with the vehicle. The data were analyzed using GraphPad Prism Software version 6.02, performing one-way ANOVA analyses followed by Tukey's multiple comparison test. ^*∗*^*p* < 0.01, ^*∗∗*^*p* < 0.001, ^*∗∗∗*^*p* < 0.0001, and ^*∗∗∗∗*^*p* < 0.00001 vs. control.

As demonstrated in Figure 3, 24-hour treatment with 10 *μ*M 2-ME or 1 mM FA did not significantly increase the number of early and late apoptotic cells, while increased the number of necrosis up to 9% and 4%, respectively. Notably, combined treatment with 2-ME and FA induced both apoptosis and necrosis in A375-treated cells. We observed approximately 10% apoptotic cells and 25% necrotic cells after 24-hour combined treatment with 10 *μ*M 2-ME and 1 mM FA as compared with control cells (0.6% apoptotic cells, 3% necrotic cells, respectively) (Figure 3).

### 3.3. Nitro-Oxidative Stress Is Involved in Anticancer Mechanisms of 2-ME and FA in the Melanoma A375 Cellular Model

Due to the fact that both 2-ME and FA may regulate the level of reactive oxygen (ROS) and nitrogen species (RNS) in cancer cells [47–50], we evaluated the effect of the compounds on pool of nitro-oxidative stress in the melanoma A375 cellular model by means of flow cytometry. First of all, we performed the experiments using DCF-DA staining to determine the level of reactive oxygen species [51].

**Figure 4:**
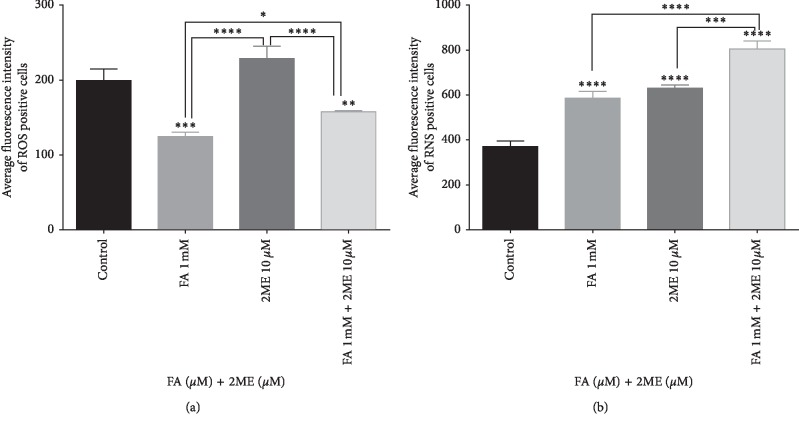
(a) Mean fluorescence intensity of ROS positive cells after 8 hours incubation of A375 line cells with 10 *μ*M 2-ME and 1 mM FA separately or in combination. (b) Mean fluorescence intensity of RNS after 8 hours incubation of A375 line cells with 10 *μ*M 2-ME and 1 mM FA separately or in combination. Values are the mean ± SE from three independent experiments. No error bar means the thickness of the line is greater than the error. ^*∗*^*p* < 0.01 compared with the vehicle. The data were analyzed using GraphPad Prism Software version 6.02, performing one-way ANOVA analyses followed by Tukey's multiple comparison test. ^*∗*^*p* < 0.01, ^*∗∗*^*p* < 0.001, ^*∗∗∗*^*p* < 0.0001, and ^*∗∗∗∗*^*p* < 0.00001 vs. Control.

As demonstrated in Figure 4(a), 8-hour treatment with 1 mM FA reduced the level of DCF-DA-stained cells which confirms its antioxidant properties. On the other hand, separate 8-hour treatment with 10 *μ*M 2-ME increased the level of oxidative stress in melanoma A375 cells. Notably, FA scavenged the 2-ME-generated oxidative stress in our experimental model (Figure 4(a)).

We have previously evidenced that one of the anticancer modes of 2-ME is associated with a selective increase in the nitric oxide level [8, 9, 46, 47]. Therefore, in the next part of the study, we aimed to determine the impact of compounds on changes within the level of nitric oxide in melanoma A375 cells via DAF-DA staining [8, 52, 53]. Notably, herein, we evidenced that induction of nitric oxide after 8-hour treatment with 10 *μ*M 2-ME can be also extended to the melanoma A375 cellular model (Figure 4(b)). We further evaluated that separate 8-hour treatment with 1 mM FA either increased the level of nitric oxide in the established experimental model. Interestingly, combined 8-hour treatment with 2-ME and FA significantly increases the level of free radical as compared to separate treatment with both compounds (Figure 4(b)). This result may suggest an observed synergistic effect between 2-ME and FA in melanoma cells.

### 3.4. Effect of 2-ME and FA on the Level of Major Hsps: Hsp60, Hsp70, and Hsp90 in the Melanoma A375 Cellular Model

Due to the fact that Hsps may be considered as the indicators and biomarkers of nitro-oxidative stress, we determined the impact of both 2-ME and FA on the level of Hsp60, Hsp70, and Hsp90 by means of western blotting analyses.

**Figure 5:**
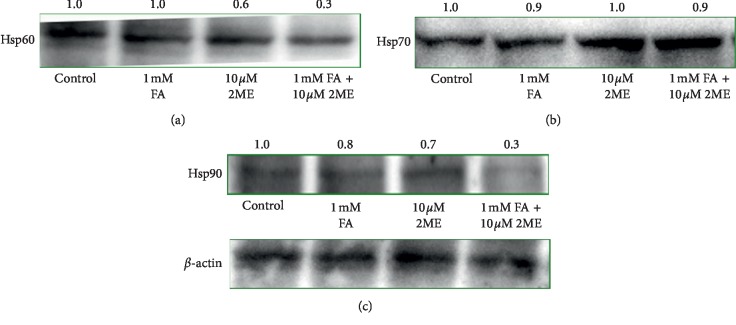
(a) Impact of separate and combined 24-hour treatments with 10 *μ*M 2-ME and 1 mM FA on Hsp60 protein expression in A375 cells evaluated by western blotting. (b) Impact of separate and combined 24-hour treatments with 10 *μ*M 2-ME and 1 mM FA on Hsp70 protein expression in A375 cells evaluated by western blotting. (c) Impact of separate and combined 24-hour treatments with 10 *μ*M 2-ME and 1 mM FA on Hsp90 protein expression in A375 cells evaluated by western blotting. Densitometric analysis of HSP/beta-actin was performed using Quantity One 4.5.2 software. The representative images are shown.

At the outset, we evaluated the influence of the 24-hour treatment with 10 *μ*M 2-ME and 1 mM FA, separately or in combination, on the Hsp60 protein level. As presented in Figure 5(a), western blot analyses of Hsp60 indicate a decrease in Hsp60 protein level by 35% and 65% relative to the control, after separate treatment with 10 *μ*M 2-ME and after combined treatment with 10 *μ*M 2 ME and 1 mM FA in melanoma A375 cells, respectively. The Hsp60 protein level was not significantly changed after combined treatment with separate treatment with 1 mM FA.

Subsequently, the changes within Hsp70 protein level were investigated in the melanoma A375 cellular model (Figure 5(b)). Notably, the obtained results indicate no changes in Hsp70 protein level as compared to the control after 24-hour treatment with both 10 *μ*M 2-ME and 1 mM FA separately or in combination.

Furthermore, our western blot analyses indicate a decrease in Hsp90 protein expression in the A375 cell line by 13%, 29%, and 69% relative to the control, after 24-hour treatment with 1 mM FA and 10 *μ*M 2 ME separately and in combination, respectively (Figure 5(c)).

## 4. Discussion

In the current study, we presented the anticancer potential of 2-ME in the melanoma cellular model. Previously, the efficacy of 2-ME towards melanoma cells was investigated in both *in vitro* and *in vivo* studies [12, 27, 28, 54]. 2-ME has pleiotropic activity in cancer cells. Interestingly, 2-ME suppresses the glycolytic state of melanoma 435R cells [27]. Moreover, 2-ME treatment decreases pRb and cyclin B1 expression, increases p21/Cip1 expression, and induces G2/M cell cycle arrest in both 2D and 3D melanoma cellular models [12].

Notably, employed in our studies a natural compound, FA, has anticancer potential and even enhanced anticancer activity of 2-ME in melanoma cells. It is suggested that phenolic compounds generally maintain normal homeostasis by inducing apoptosis in various tumour cells [55]. Many studies investigated cytotoxic and proapoptotic effects of polyphenols in various cancers [36, 50, 56, 57]. In consistency with our outcomes, Park et al. established the anticancer potential of FA in the mouse B16F10 melanoma cells [58]. Furthermore, Khanduja et al. proved that phenolic compounds, such as FA, significantly reduce apoptosis in normal peripheral blood mononuclear cells, which suggests limited cytotoxicity of FA [59]. Even more importantly, the significant role of FA in the prevention of skin cancer was also proved [57].

The cytotoxic activity of both compounds seems to be strictly associated with induction of nitro-oxidative stress. In our previous studies, we evidenced that 2-ME selectively upregulates neuronal nitric oxide synthase which results in generation of nitric oxide in cancer cells [8]. Herein, indeed we observed increased level of nitric oxide both after treatment with FA and 2-ME. This effect was even enhanced after combined treatment with the compounds. The mechanism of induction of nitric oxide by FA in cancer cells still needs to be evaluated. Nonetheless, FA was reported to generate nitric oxide through upregulation of argininosuccinate synthase in inflammatory human endothelial cells [60]. On the other hand, FA inhibits nitric oxide production and inducible nitric oxide synthase expression in rat primary astrocytes [61].

Notably, in contrast to altered nitric oxide induction, FA scavenged ROS in our melanoma experimental model. The compounds were also able to reverse 2-ME induction of ROS. These results confirm antioxidant properties of FA. However, the observed contradictory effect of 2-ME and FA on ROS may result in protective role of FA against cytotoxicity of 2-ME. Indeed, the protective role of FA against cisplatin-induced ototoxicity was previously demonstrated [62]. FA was also reported to protect against methotrexate nephrotoxicity [63].

Herein, we presented the involvement of major Hsps namely Hsp60, Hsp70, and Hsp90 in the modes of action of 2-ME and FA in the melanoma A375 cellular model. Notably, these Hsps seem to be also responsible for the mechanism of interaction between both compounds. To this date, there are only a few studies considering the role of Hsps in anticancer mechanism of action of 2-ME [64–67], while no one conducted on the melanoma experimental model. Similarly, there are only limited data investigating Hsps in FA mechanism [68, 69]. Depending on their localization and expression Hsps may have a dichotomal effect in cancer biology. The 60 kDa heat shock protein (Hsp60) is classically known as a mitochondrial chaperonin protein. However, accumulating data support that it is localized in extramitochondrial compartments as well [70–75]. As a primary mitochondrial chaperone, Hsp60 is essential for mitochondrial protein homeostasis [76]. However, it is also implicated in the cell survival and apoptosis signaling pathways [41]. Increased protein level of Hsp60 has been detected in various malignant cells including colon [77], cervix [78], prostate [79], or melanoma [80]. In many of the cases examined, higher expression was correlated with poorer prognosis [81–83].

In consistency with these studies, our obtained outcomes indicate that 2-ME decreased Hsp90 protein level in melanoma cells. To this date, there are no data considering the role of FA in regulation of Hsp60 protein level. Although FA itself does not affect the Hsp60 protein level in melanoma cells, it enhances the activity of 2-ME to decrease Hsp60 expression. These data indeed are strictly associated with the level of apoptotic and necrotic cells as well as concentration of nitric oxide in melanoma 2-ME and FA-treated cells. On the other hand, 2-ME was reported to increase Hsp60 protein level in estrogen-positive breast adenocarcinoma MCF-7 cells [66]. Thus, the role of expression of Hsp60 seems not to be clear and to depend on an experimental model, i.e., type of cancer cells. Indeed, higher expression of Hsp60 was observed in early-stage ovarian cancer than advanced-stage in one other report [84]. It was further investigated that increased expression of Hsp60 is correlated with higher susceptibility of melanoma cells to immune chemotherapy [85].

Targeting Hsp70, beyond Hsp60, is a new therapeutic approach. Most compounds are active Hsp90/Hsp70 inhibitors and induce cancer cell death [86]. Hsp70 directly or indirectly modulates the intrinsic and extrinsic apoptotic pathways. Inhibition or knockdown of Hsp70 increases sensitivity of cells to apoptosis [87, 88]. Human cells produce high levels of Hsp70, constitutively expressed as Hsc70, mitochondrial Hsp75, and GRP78, which are found in the endoplasmic reticulum [86]. Under nonstressed conditions, cells express constitutive levels of Hsp70. However, their enhanced expression, a feature of cancerous or stressed cells, increases survival of these cells. Clinical studies indicate that increased expression of Hsp70 is associated with tumorigenesis, poor prognosis, and chemoresistance of numerous malignancies, including melanoma [86, 89, 90]. Notably, in our melanoma experimental model, changes in Hsp70 were not observed after treatment with 2-ME and FA separately or in combination. It is important, especially for mechanisms of chemoresistance, as accumulation of Hsp70 reduces the induction of cancer cell death, thus decreasing the antitumour efficacy [86].

Due to the fact that Hsp90 forms a chaperone machinery with Hsp70, we have established the impact of 2-ME and FA on this protein. Hsp90 is an interesting target for cancer therapy because it is involved in folding and stabilization of numerous proteins, including those that contribute to the development of cancer. In mammals, Hsp90 chaperones include Hsp90 alpha and Hsp90 beta, GRP94 (94 kDa glucose-regulated protein), and TRAP-1 (tumour necrosis factor receptor-associated protein 1) localized in the cytoplasm, ER, and mitochondria, respectively [91]. Hsp90 is implicated in the pathogenesis of numerous diseases, including cancer. Several cancer proteins depend on Hsp90 machinery and chaperones for their folding and maturation, i.e., steroid hormone receptors and transcription factors [91]. Therefore, pronounced expression of Hsp90 has been detected in almost all types of cancers, including melanoma [44, 92, 93]. Hsp90 expression is higher in metastatic melanoma and associated with malignant features as Clarke's level in cutaneous melanoma and larger tumour size in uveal melanoma [44]. Herein, we evidenced that both FA and 2-ME downregulate the Hsp90 expression, this effect is even enhanced after combined treatment with the compounds. These data are consistent with increased anticancer efficacy of combination of compounds in relation to separate treatments. Up to date, there are only few data about the role of Hsp90 in anticancer mode of action of 2-ME. Chauhan and coworkers evidenced that downregulation of Hsp90 gene expression via 2-ME is a mechanism of overcoming the chemoresistance [94]. On the other side, Kim et al. established upregulation of Hsp90 alpha in breast cancer MCF-7 adenocarcinoma cells [66]. To this date, there are no studies about the role of Hsp90 in anticancer mode of action of FA. Nonetheless, it was hypothesized that antidepressant-like effect of FA is associated with activation of MAPK kinases pathway and Hsp90 [95, 96]. These contradictory results may be explained by different experimental models (cancer and nontransformed cells) as well as experimental conditions, i.e., time of incubation.

## 5. Conclusions

Herein, we presented a synergism between a potent anticancer compound, 2-ME, and a naturally occurring polyphenol, FA. The molecular mechanism of observed interaction is at least partially associated with downregulation of Hsp60 and Hsp90 and induction of nitric oxide in the melanoma A375 cellular model. Furthermore, scavenging of 2-ME-induced ROS by FA may be a protective mechanism against enhanced toxicity of 2-ME. Therefore, further investigation of sources of nitro-oxidative stress in 2-ME and FA-treated cells is still needed. Nonetheless, the obtained data strongly support the anticancer effect of 2-ME and FA and their potential role in adjuvant chemotherapy.

## Data Availability

The data used to support the findings of this study are available from the corresponding author upon request.
